# CUSP: an algorithm to distinguish structurally conserved and unconserved regions in protein domain alignments and its application in the study of large length variations

**DOI:** 10.1186/1472-6807-8-28

**Published:** 2008-05-31

**Authors:** Sankaran Sandhya, Barah Pankaj, Madabosse Kande Govind, Bernard Offmann, Narayanaswamy Srinivasan, Ramanathan Sowdhamini

**Affiliations:** 1 National Centre for Biological Sciences (TIFR), UAS-GKVK Campus, Bellary Road, Bangalore 560 065, India; 2 Mathematical modeling and Computational Biology group, Centre for Cellular and Molecular Biology, Hyderabad, India; 3 Laboratoire de Biochimie et Génétique Moléculaire, Université de La Réunion, La Réunion, France; 4 Molecular Biophysics Unit, Indian Institute of Science, Bangalore, India

## Abstract

**Background:**

Distantly related proteins adopt and retain similar structural scaffolds despite length variations that could be as much as two-fold in some protein superfamilies. In this paper, we describe an analysis of indel regions that accommodate length variations amongst related proteins. We have developed an algorithm CUSP, to examine multi-membered PASS2 superfamily alignments to identify indel regions in an automated manner. Further, we have used the method to characterize the length, structural type and biochemical features of indels in related protein domains.

**Results:**

CUSP, examines protein domain structural alignments to distinguish regions of conserved structure common to related proteins from structurally unconserved regions that vary in length and type of structure. On a non-redundant dataset of 353 domain superfamily alignments from PASS2, we find that 'length- deviant' protein superfamilies show > 30% length variation from their average domain length. 60% of additional lengths that occur in indels are short-length structures (< 5 residues) while 6% of indels are > 15 residues in length. Structural types in indels also show class-specific trends.

**Conclusion:**

The extent of length variation varies across different superfamilies and indels show class-specific trends for preferred lengths and structural types. Such indels of different lengths even within a single protein domain superfamily could have structural and functional consequences that drive their selection, underlying their importance in similarity detection and computational modelling. The availability of systematic algorithms, like CUSP, should enable decision making in a domain superfamily-specific manner.

## Background

Protein databanks such as the PDB [1], with nearly 47,000 structures in the current year, are growing at a rapid pace. Interestingly, the increase in the number of protein structures in the last decade is not accompanied by a concomitant rise in the number of novel folds. This suggests that protein folds are resilient to exploit their large degrees of conformational freedom and can tolerate large modifications in sequence and length. Structural comparisons of related proteins show that changes, in the form of substitutions, deletions or insertions are accommodated into existing protein scaffolds. Protein domains show from two-three residue variation to over two-fold length variations as in the PDB entries for P-loop NTP hydrolases and the TIM fold.

Recent studies correlating domain length variations with the taxonomy spans of domains report that over one-third of all domains tend to increase/decrease in domain size. The fraction of domains that increase in domain size is two-fold larger than domains that decrease in size[2]. Analysis of protein length distributions across the main kingdoms have also shown that mean protein lengths are 40–60% greater in eukaryotes than in prokaryotes[3]. Such expansions in length correlate with the accretion of functional motifs during the evolution of sophisticated regulation networks in higher eukaryotes.

Structural variation is influenced by the number, length and location of insertions and deletions of residues (indels) [4]. Pascarella and Argos [5], noted that less than 2% of indels are longer than 10 residues suggesting that a gradual accretion of protein length through shorter indels can achieve structural diversity. Reeves and co-workers [6], in an analysis of domain variations in CATH superfamilies have shown that even at low sequence identities (< 30%), 50% of the domain structure is conserved. However, changes in the form of structural re-orientations and the number of structural elements are high between remotely related proteins. Domain length variations although discontinuous in sequence co-locate in 3D space and mediate functional variety.

In a separate analysis on the study of physical parameters between related domains of a superfamily, "structural templates" were shown to have a strong correlation of physical parameters such as solvent accessibility, hydrogen bonding patterns, spatial orientations and interactions between different members [7]. Such segmental conservation of features suggests that such features are not as well preserved in poorly conserved regions resulting in structural and functional diversity amongst related domains through variable regions. Length accretions are critical in mediating structural and functional variety in proteins and it is, therefore, important to understand their properties and determine if class-distinct trends operate on protein domains. We extend earlier analysis on indel properties further by annotating such regions in terms of their preferred structural types, lengths and biochemical parameters and look for class-specific trends, if any. Such indels also extend functional and structural support to protein domains and this is also discussed briefly for a few superfamilies.

We report an algorithm, CUSP, which identifies conserved units of structure in proteins and distinguishes such regions from indels where length variations are introduced. The PASS2 database[4] provides structure-based alignments of non-redundant representatives of protein superfamilies sharing < 40% sequence identity. Since initial equivalences are specified using STAMP 4.0[8] or LSQMAN[9], such alignments maximize structurally similar regions amongst related domains and distinguish them from indels that are structurally variable across different members. These alignments derived through COMPARER[10] have examined protein domains that show not only low sequence conservation but also demonstrate variety in length and thus serve as ideal starting points to describe indel regions.

CUSP was used to examine length variations in 353 multi-membered superfamily alignments (> 3 sequence diverse relatives) from the PASS2 database [4]. To determine if observed trends are affected by the inclusion of more proteins, sequence homologues of the structural entries in PASS2 were included from the GenDis database[11]. In a separate analysis, the CUSP algorithm was also applied to such alignments to detect core features of a domain superfamily. Further, we have extended the study to analyze the conservation of a biochemical property such as solvent exposure in structurally conserved and unconserved regions.

## Methods

### Dataset

353 structure-based superfamily alignments from PASS2 database [4], with more than 3 members, at < 40% identity cutoff and nearly equal representation from the four major structural classes (72, 81, 88 and 112 superfamilies from α, β, α/β (AorB) and α+β (AplusB) classes respectively) were considered for the analysis. Since PASS2 derives from SCOP hierarchical schemes[12], only non-redundant representatives are considered in the alignments and biases due to over-representation of similar structures are avoided.

### CUSP: Detection of conserved units of structure in protein structural alignments

**Figure 1:**
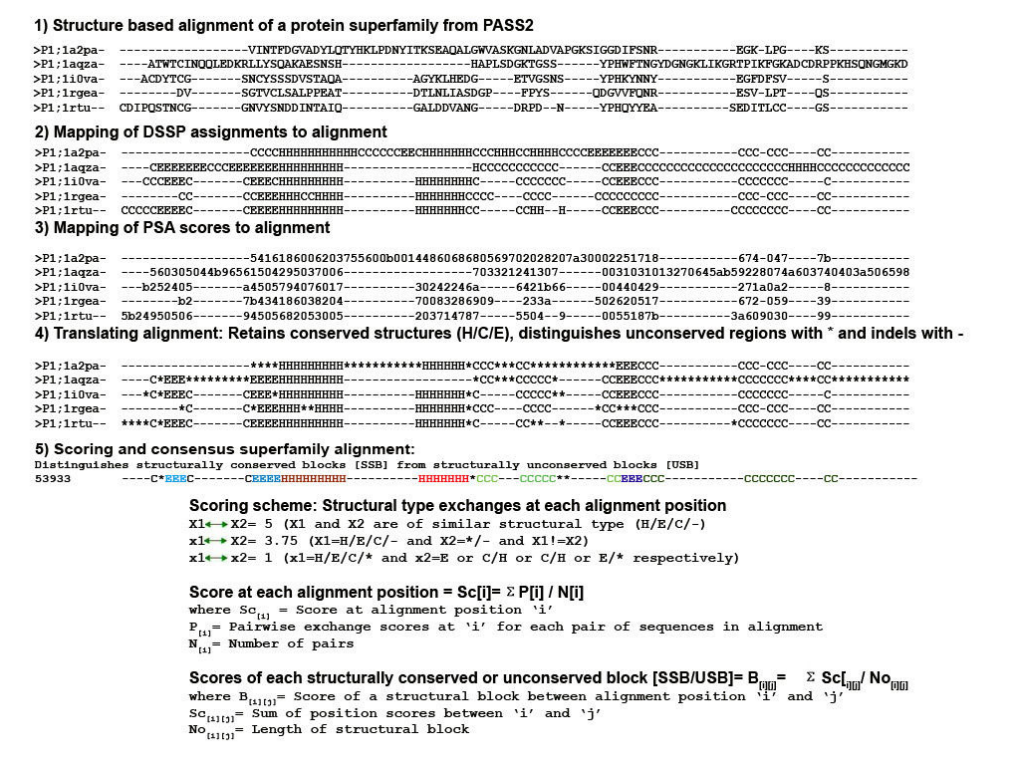
**Schema of CUSP algorithm and the scoring scheme employed for identifying structurally conserved and unconserved blocks [SSB and USB]**. Steps 1–4 illustrate the steps involved in processing structure-based alignments of an example domain superfamily. Scoring schemes that capture structural type exchanges at each position in the alignment (represented as X1 and X2 exchanges for comparisons of each pair) are first applied to each position. Consecutive positions with high scores are merged to identify structurally conserved blocks and distinguish them from indels. An average score is associated with each such block and used to annotate the alignment to distinguish indel regions (USB) from 'core' regions (SSB). In the example, highly conserved structural blocks (H, E and C) identified by high block scores (> 4.5), are indicated in maroon, dark blue and dark green respectively. Conserved blocks that show 'medium' conservation are also indicated (red (helix), cyan (strand) and light green (coil)). The remaining regions are treated as USB.

Starting from PASS2 alignments [4], CUSP maps DSSP assignments of secondary structure[13] to the alignment (see Figure 1). Likewise, solvent accessibility scores computed through PSA program [14] are also mapped to every sequence. Each alignment position is scanned and the assignments H, C and E at each position are retained if such a structural type shows > 75% occurrence. This is applied to capture consensus trends observed in the majority of members as described earlier in JOY assignments [15] and in the detection of equivalent structures amongst superfamily representatives [6]. Gaps (-) are retained to account for insertions or deletions in any member. A '*' replaces a structural type at a position in the alignment when such a structural type shows < 25% occurrence at that position. To determine the consensus residue at each position, a scoring scheme that scores absolute matches of a structural type as 5, mismatches as 3.75 and gap exchanges as 1 (Figure 1), is employed. Although score assignments were chosen non-empirically, they were optimized on analyzing a large variety of alignments that varied in the number of representatives and the employed scheme was effective in differentiating strictly structurally conserved positions from indel regions. The scoring scheme was applied to score all exchanges between observed structural types at each position (Figure 1: described as X1–X2 exchanges) and an average score (Figure 1: Sc_[i]_) and a consensus structural type was assigned to every alignment position. Next, consecutive alignment positions of identical structural types were merged to form a structural block and a Block score, an average of the sum of scores at each position, was associated with every block (Figure 1: Block_score_[i] [j]_). Thus, a block represents a consensus unit that is conserved in all members in the alignment.

In a similar manner, average solvent accessibility scores were also associated with each structural block (not shown in the figure). Since the score is averaged over each position in the alignment, the block score is indicative of the extent of conservation of each structural type (H, C, E or -) in each block. Each structural block was associated with the tags 'Poor' (block score < 3), medium (block score 3–4.5) and high (block score 4.5–5.0). Finally, a consensus structural alignment is derived for the protein superfamily that not only delineates the structurally conserved blocks (SSB: H, E or C) from structurally unconserved blocks (USB: *, -) but also annotates such regions based on block scores as 'high, medium or poor' to indicate degree of conservation.

### Validation of the algorithm and scoring schemes

The scoring scheme that we have employed was arrived at after examining domain superfamily alignments that varied in the number of representative members in the alignment. Although it is well appreciated that different approaches produce quite different alignments[16], structural alignments of ten superfamilies, derived independently using other alignment methods such as CE[17] and CDD[18], were also tested with the CUSP scoring scheme. Primarily, we wanted to determine if the applied scores were robust in identifying structurally conserved features in related domains. The number of structurally equivalent positions reported by either method was obtained and compared with the number of 'core' conserved residues identified by the CUSP scoring scheme when applied to domain superfamily alignments from PASS2. In each of the superfamilies considered, the CUSP scoring scheme was robust in capturing strictly conserved features. Specifically, inherent biases in the superfamily, for instance, the conservation of a minimum of 4 helices in the cytochrome C superfamily, could be captured independent of the alignment method and to that extent the schemes employed are predictive and can describe the strictly conserved features of a superfamily.

### Length variation in protein superfamilies

Mean domain sizes for each superfamily were determined by averaging over the lengths of individual members. Standard deviations in length from the mean domain sizes were calculated for every member using standard formula and averaged for the entire superfamily. The degree of length variation for every member from the mean domain size of its superfamily was calculated by expressing as a ratio the length difference of each member to its mean domain size.

$$\text{Length difference}=\frac{\left|l_{i}-M\right|}{M}*100$$

where *l*_*i *_= length of a protein superfamily member

*M *= Mean domain size of each superfamily

**Figure 2:**
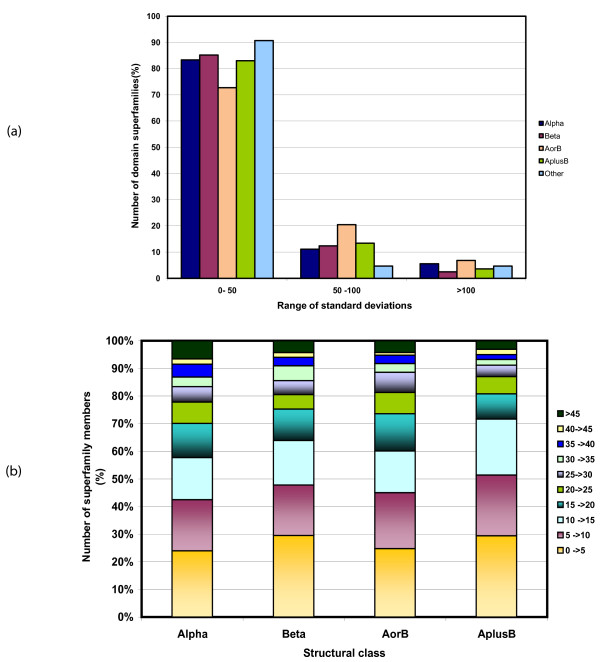
**a) Distribution of length variation (described by mean standard deviation) in 353 domain superfamily members of Alpha, Beta, Alpha/Beta (AorB) and Alpha +Beta (AplusB) classes.** b) Class specific distribution of the extent of length variation (expressed as a ratio of mean domain size) of all superfamily members.

The distribution of length differences of the ~2500 proteins in the 353 superfamilies was plotted. Superfamilies in which a majority of the members (> 75%) show > 30% length variation were considered as "length-deviant" superfamilies and others with less than 10% variation in length from the mean domain size were considered as "length-rigid" superfamilies. The extent of deviation and the number of members that distributed in various length ranges were both employed in the distinction of superfamilies as length-rigid and length-deviant (also see Additional information Section I: S1–S5, Figures 2 and S1, and Results later).

### Application of CUSP on diverse folds and functional implications of indels

Structurally conserved features of several domain superfamilies, by careful examinations of multiple alignments, have been studied in detail in the past and are available in literature. We have applied CUSP to a few classical domain superfamilies such as globin, ferritin and cytochrome C domain superfamilies (see Additional information) to determine CUSPs performance in identifying core regions and in distinguishing indel regions in these well characterized folds. In addition, other structure alignment methods were also applied to such folds. The functional and structural implications of indel regions detected by CUSP were also examined.

## Results and Discussion

### Extent of length variation in protein domain superfamilies

Length variations in protein domains are universal and observed in all protein classes. Figure 2a shows that domain superfamilies from all classes show long length variations (> 50 residue standard deviation from the average domain size). ~20% of protein superfamilies from the α/β class exhibit > 50 residue deviations in domain length. ~70% of the superfamily members in all classes show < 50 residue deviation (Figure 2a). The extent of length variation in different multi-membered superfamilies from all classes ranges from 5 to > 45% of the mean domain size (Figure 2b) and has been used to distinguish length-rigid from length deviant domain superfamilies (Table 1). These trends are also observed on consideration of the 64 length-deviant domain superfamilies alone. For 50 of the length-deviant domain superfamilies, we observe that < 20% of the members show < 5 residue variation (Figure S1b).

**Table 1:** List of length-rigid and length-deviant domain superfamilies. This list is shown only for helix-rich class. Please look into Additional Tables 1 and 2 for full list. *Highly populated domain superfamilies (> 10 numbers).

**a) List of 'Length-rigid superfamilies' (> 4 members).**					
**S.No**	**Class**	**No_members**	**Average domain size**	**Sequence Identity**	**Description**

				**(%)**	
1	α	8	417	21	Cytochrome P450
2	α	6	323	14	Terpenoid synthases
3	α	8	250	25	Nuclear receptor ligand-binding domain
4	α	5	204	23	DNA-glycosylase
5	α	5	114	26	Calponin-homology domain, CH-domain

**b) List of 'Length-deviant superfamilies' (> 4 members).**					

*6	α	22	101	24	Cytochrome c
*7	α	32	64	26	Homeodomain-like
* 8	α	48	88	21	Winged helix" DNA-binding domain
9	α	6	92	32	C-terminal effector domain of bipartite response regulator
10	α	5	90	27	Putative DNA-binding domain
*11	α	12	88	29	Histone-fold
*12	α	12	259	17	Ferritin-like
*13	α	22	142	18	4-helical cytokines
*14	α	35	125	23	EF-hand
15	α	5	75	33	Met repressor-like
16	α	6	76	37	IHF-like DNA-binding proteins
17	α	6	191	22	6-phosphogluconate dehydrogenase C-terminal domain-like
18	α	6	308	18	Terpenoid cylases/Protein prenyltransferases
19	α	9	369	17	ARM repeat
20	α	9	202	21	TPR-like

### CUSP assignments of structurally conserved and unconserved blocks in proteins

Structural modifications, it is observed, can form extensions of pre-existing structures or insert as new structural elements in the middle of domains. Such insertions although not contiguous in sequence may lie close to each other in structure and even form sub-domain like structures. Alternately, they may accrue as additional regular (α-helix, β-strand) and irregular structures (coils) at the N and C terminal ends (Table S2). Since CUSP delineates protein alignments into structurally conserved regions and unconserved regions, it would be useful to identify if a selection principle is operational in identifying where structural modifications, because of additional lengths, can occur in related protein domains.

### Extent of length variation accommodated in SSB and USB

80% of length variations in length-deviant superfamilies from all classes are observed in USB regions with some superfamilies from the α/β class accommodating a wider range of length variation (Additional information, Section I: S2 – S4). Truncated structural elements account for 10% of these length differences (Additional Figure S1a).

### Structural characteristics and lengths of 'indels'

**Figure 3:**
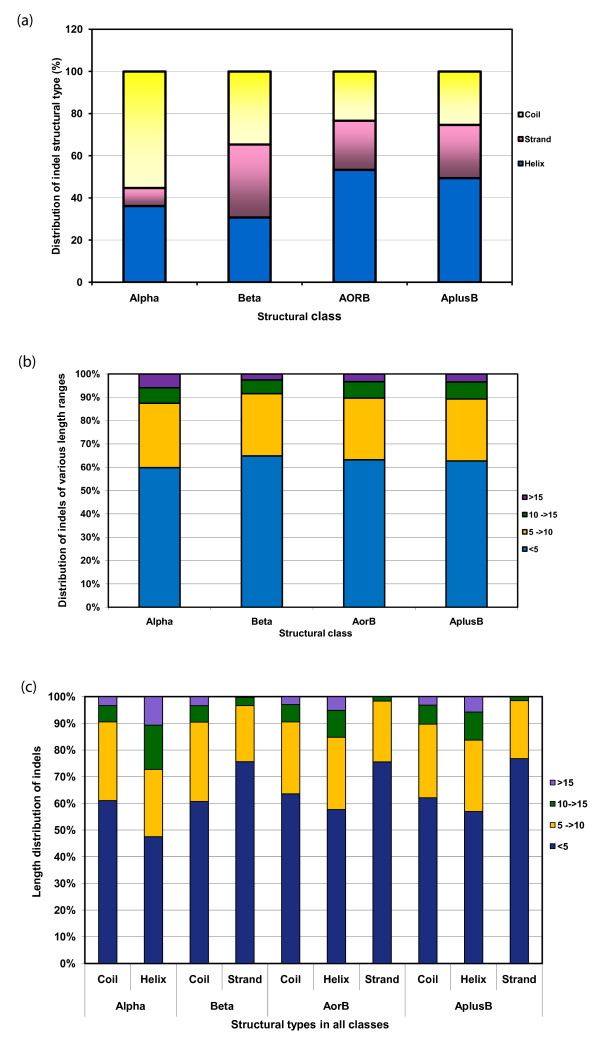
**a) Class specific distribution of the type of structure observed in indel regions. **b) Class specific distribution of indel lengths. c) Distribution of indel lengths of various structural types [α-helix, β-strand, coils] in indel regions.

**Figure 4:**
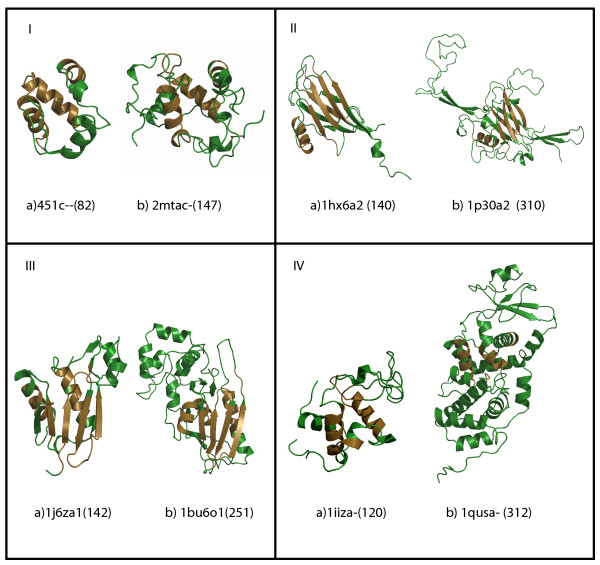
**Length adjustments in length-deviant superfamilies from the four major classes**. Panels' I-IV depict 'dwarf and giant' representative members (left and right respectively) of a deviant superfamily from alpha, beta, alpha/beta and alpha +beta class. Representative members are indicated with PDB id and domain length. CUSP reported structurally conserved regions (SSB), whose lengths and structural type are retained across all domain superfamily members (in brown), are distinguished from unconserved regions/indels (USB, in green). (a) Cytochrome C superfamily 'giant' members are 56% more likely to adjust extra length as coils and short length helices.(b) Viral proteins from β-class have acquired additional strands and coils in indel regions. Up to two-fold length variations are seen as additional coils and helices in (c) Actin-like ATPase and (d) Lysozyme-like domain superfamilies.

We have examined the nature and lengths of secondary structures that appear in indels. Here, we find that regular secondary structures such as helices and strands are observed in indels, in addition to coils (Figure 3a). In fact, a class-specific trend emerges in the present analysis (Figures 3a and 4) and we find that additional length between related proteins is accrued in ~50% of protein superfamilies of the α-β (α/β or α+β) class as α-helices. Examination of protein structures of representative 'giant' and 'dwarf' members of length-deviant protein superfamilies confirm these trends. 56% of protein superfamilies from the α-class such as the Cytochrome C (Figure 4a) show additional coils in indels while β-class proteins introduce either additional β-strands/coils (Figure 4b). The giant and dwarf members of the Actin-like ATPase and Lysozyme superfamilies (Figure 4c and 4d) accommodate up to two-fold variation in length primarily as additional helices and coils. On consideration of the 64 length-deviant superfamilies alone, similar trends are obtained and helices and coils are both highly favored in indels (Figure S1c). For 70% of the highly populated length-deviant domain superfamilies (Table 1), nearly 40% of indels are coils (Figure S1d). Manual examination of the locations of these additional structures shows that such indels can either act as extensions of previous secondary structures or occur as insertions in the middle of domains. Some of these insertions extend from the N and C terminal domains and in superfamilies such as the SAM domain and lysozyme (Figures 4c and 4d), such indels are long and sub-domain like.

The length distributions of such indels in different classes also show interesting trends. We find that 60% of indels are < 5 residues (Figure 3b). Medium-sized indels of between 5–10 residues are noticed in 20% of all indels in the dataset. Only 6% of all indels are found to be > 15 residues in length. Similar trends were also observed in earlier analysis on homologous superfamilies [5,6] although on smaller and different datasets.

45% of the additional α-helices in indels of helix-rich length-deviant superfamilies are shorter than 5 residues (Figure 3c). A majority of the α-helices appearing in USB regions of β and α-β protein superfamilies are < 5 residues although in all superfamilies, longer α-helices (between 5 and 15 residues) are also observed (~20%). ~70% of indels appearing as β-strands are short length (< 5 residues) and this may relate to the cost involved in satisfying the inherent nature of β-strands to form sheets. Additional strands of longer length (> 10 residues) are observed in fewer than 5% of all length-deviant β-rich superfamilies. Such strands in indels could be extensions of pre-existing strands or occur as shorter length β-hairpins and, therefore, strands longer than 15 residues are not noticed in indel regions.

We observe that percentage variation in terms of the total number of α-helices, coils and β-strands is more in length-deviant superfamilies than in length-rigid superfamilies (see Additional information, Section I: S1, and Tables S4 and S5). In some of the length-deviant superfamilies (Additional Tables S2 and S5), the number of additional structures is large enough to form domain like structures.

Manual examination of the structural alignments of the giant and dwarf domains of length-deviant domain superfamilies shows that in all classes, the accretion of single, long secondary structures is less common and instead many short length indels are arranged to form super secondary structural motifs (Table S2). Thus, isolated or solvent-exposed extra secondary structures are avoided and additional units confer structural or functional support in each domain superfamily. In order to address if these trends are observed after including immediate sequence homologues of these superfamiles, we consulted the pre-curated results from GenDis database [11] for the top-five length-deviant and length-rigid superfamilies belonging to the four major structural classes. For each superfamily, between 250 to 800 sequence homologues were considered for assessing trends in length variation. We find similar trends of length variation in the superfamilies distinguished as "length-rigid" and "length-deviant" using structural homologues alone, even on the inclusion of sequence homologues in these superfamilies (data not shown). This suggests that superfamilies identified as length-deviant/rigid are likely to remain so even with the availability of more structures.

### Solvent accessibility in conserved structural blocks

The conservation of a biochemical property such as solvent accessibility in regions annotated as SSB and USB was analyzed (see Additional information, S5) to determine if such regions behaved distinctly from each other. Additional Figure S2a shows that in Beta class superfamilies, structurally unconserved regions (USB) arising from indels or structural replacements are usually exposed to the solvent. Amongst these, in structurally conserved regions (SSB), β-strands show a distinct preference for avoiding solvent while coils and α-helices are partially/well- exposed to the solvent. Likewise, Additional Figure S2b shows the distribution of the average PSA scores in different types of structurally conserved blocks in all classes. (Additional Figures S3–S5 show trends in these parameters for other classes).

### Application of CUSP algorithm in the identification of structural scaffolds

The CUSP algorithm examines structure-derived alignments to delineate structurally conserved regions from structurally variable regions in protein domain superfamilies. Thus, applications of the algorithm on domain superfamily alignments are well capable of identifying 'core' regions, common to all members, from 'variant' regions. In order to verify this, we have examined the scores assigned to various conserved blocks identified by the program on some well characterized folds such as the Globins, Ferritins and Cytochrome C (see Additional information). Each of these folds is known to show considerable variations in length that are accommodated as indels.

### Globins

**Figure 5:**
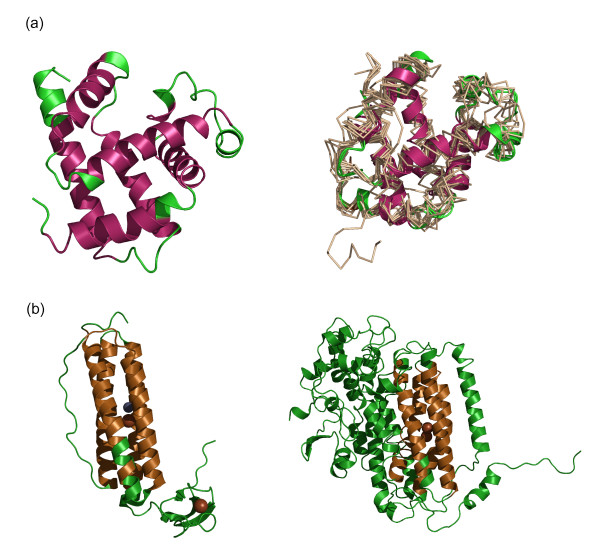
**a Structurally conserved regions identified in the globin fold (in pink) by CUSP on independently derived alignments from PASS2 and CE (left and right respectively).b **Dwarf and giant domains in the Ferritin superfamily ([1dvba1 (1–147)] and [1mtyd- (15–526)], left and right respectively) show a common conserved core of 4 helices (in brown) surrounding a central Fe atom. Additional lengths in methane monoxygenase hydroxylase, the giant domain, (in green) participate in domain interactions.

The three-dimensional structures of globins are known, from crystallographic analyses, to be very similar. In an earlier analysis of the conserved features of this fold involving 226 sequences, it has been shown that the globin family of proteins differ greatly in their amino acid sequences and conserve only two residues in all sequences. Residue identities of some pairs of sequences are even as low as 16% [19]. Structure-guided alignments generated for these proteins have shown that although individual chains vary in size between 132 and 157 residues, only 102 residue sites are common to all globins due to many deletions and insertions. These sites form six separate regions that lie in the core conserved helices. Insertions and deletions between these regions involve separations of different lengths in different sequences. Other detailed reviews on the phylogenies and differences between constituent members universally agree on the conservation of a core fold constituted by the A, B G and H helices [20]. Functional variety is attributed to differences in the remaining structural elements. As shown in Figure 5a, the CUSP algorithm attributes high scores to such well conserved regions and recognizes the four core helices. In fact, these scores and structurally conserved blocks are also identified by using alignments involving different members and methods. For the same superfamily, we have examined the alignments generated by CE [17] and CDD[18]. Irrespective of the number of sequences and the alignment algorithm applied to align the sequences, CUSP is seen to detect the structural scaffold involving the core A, B, G, H helices to a high accuracy. Figure 5a and Table S3 show that in the globin domain superfamily, CUSP reports 107 residues as structurally equivalent, CE derived alignments treat 119 residues as structurally conserved while CDD reports 84 residues as strictly conserved.

### Ferritin like superfamily

This superfamily of the alpha-rich fold includes members that are di-iron carboxylate proteins. The average domain size of the superfamily is 250 residues and includes small domains such as ruberythrin (1dvba1, 147 residues) and giant domains such as methane monoxygenase hydroxylase/MMO (1mtyd,512 residues). The two domains catalyze dioxygen-dependent oxidation-hydroxylation reactions[21]. All members are characterized by the presence of a duplicated motif consisting of two consecutive helices. An iron-coordinating glutamic or aspartic acid is located in the first helix and there is an EXXH (single-letter code for amino acids) motif in the second, but there are no other obvious sequence homologies. CUSP when applied to structure-based alignments for the domain superfamily detects the consecutive helices that strictly co-ordinate Fe (Figure 5b). These conserved helices, in fact, typify the conserved scaffold of the domain superfamily and are also detected from independently derived CE alignments of Ferritin domains. As seen in Table S3, both CE and CUSP agree well on the number of structurally equivalent residues for the domain superfamily. Large difference in size that occurs as additional helices and several loops are found to be associated with the number of interacting domains in the giant member MMO which is far more than ruberythrin. This difference could account for the acquisition of extra structural elements that can interact with different domains.

### Role of indels in structural and functional diversity

**Figure 6:**
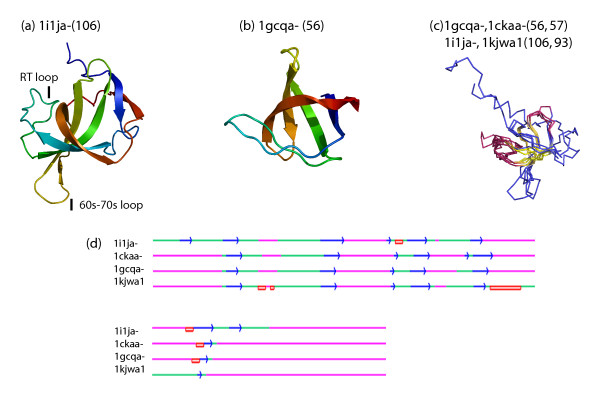
Giant and dwarf domains of the SH3 domain like superfamily (a) [1i1ja(1–106)] and (b) [1gcqa-(158–213)]) show additional structures near the ligand-binding site. Structural superposition of the domain superfamily members (c) shows an appreciable conservation of the core structures (in yellow). (d) Structview representation of the alignment of different domain members of the protein superfamily shows a well conserved core involving β-strands and indels acquiring secondary structure in the giant domain.

Indels, irrespective of their location, seem to confer a structural or functional variation to the domain superfamily in which they occur (Table S2). For instance, the members of the SH3 domain superfamily (Src Homology) show up to two-fold length variation. This is a family of molecular modules that is conserved amongst diverse proteins which function in protein-protein interactions for intracellular signal transduction. These interactions are effected through the recognition of a short proline-rich sequence, that adopts a left-handed polyproline type II helical conformation, embedded in proteins [22]. A giant member of this domain superfamily, MIA (Melanoma inhibitory activity protein), differs from the typical members in structural and functional aspects. In contrast to the typical members that are intracellular and modular, MIA is a single domain, extracellular protein. Here, additional lengths are not only seen as N and C terminal extensions that result in wider and larger barrels (Figure 6a–c) but also as additional sheets and 3_10 _helices in the middle of the structure. Length differences extend to the RT loop and 60s–70s loops that flank the ligand binding region of SH3 domain. A superposition of four domains in this superfamily shows incremental additions to the termini and acquisition of additional structures in the middle of the domain such as in the RT and 60s–70s loops (Figure 6d). These indels mediate functional differences from the typical domain members and involve in the ability to recognize ligands other than conventional polyproline helices. Thus, in the SH3 domain superfamily, structural add-ons tune a conventional scaffold to meet new requirements of location, structure and function.

## Conclusion

In our analysis, we have estimated the extent of length variation in protein superfamilies and employed it as a measure of structural variation between homologous proteins. Numerous measures have been used to quantify protein structural similarity and these include RMSD, SSAP, contact maps, DALI and VAST scores [23-26]. We are interested in the tolerance of folds to large variations in length and have, therefore, employed standard deviation and mean length variation to determine this. Proteins of similar lengths may still differ in the orientations of individual secondary structures and adopt different folds. To that extent, a simple scoring scheme that parses pre-derived structural alignments of known related proteins from the PASS2 database and quantifies the extent of length variation in all protein superfamilies is used to empirically estimate trends emerging in the dataset. We have also performed the analysis on multi-membered domain superfamilies (> 3 members) for an empirical assessment of the data involving 353 domain superfamilies. Additionally, trends obtained in the dataset are noticed on consideration of the most length-deviant or highly populated domain superfamilies alone.

We have presented a method, CUSP, which processes protein structure-driven alignments to identify conserved structural units, common to all related proteins. In doing so, regions that allow variations to accumulate and confer uniqueness to each protein, annotated as USB, are also identified for every superfamily. The scoring schemes were arrived at after examining alignments derived independently from other approaches such as CE and CDD. In 8 of the 10 superfamilies examined, CUSP detects > 60% of the conserved residues reported by other alignment methods. For the two superfamilies which show < 45% coverage, the large difference in the number of structural entries examined may be responsible for the difference in performance. While the alignments from CDD included very close sequence homologues, the structural representatives considered in the CE alignment included domain members of similar lengths and also include more sequence diverse members. A strict cut-off of 75% is employed to characterize structural types at each alignment position as H, E or C and this in fact, increases the stringency of the scores. These assignment and scores therefore, are representative and predictive of SSB assignments in all and new sequence relatives in the superfamily. Cut-off schemes similar to ours have been employed earlier in JOY representations of structural alignments[15] and in estimating equivalences of secondary structures (SSE) for deriving matrices[27].

We have also attempted a study of the domain contexts, associations of length deviant domains and their functional consequences (Table S2, and manuscript under preparation). Reeves *et. al*., [6] have examined equivalent secondary structures between CATH superfamilies and suggest that such additional structural elements contribute effectively to functional variety in the highly populated superfamilies.

Since the CUSP algorithm works with a scoring scheme to detect consensus trends in a majority of the superfamily members, the extent of conservation of each structural type in each block is annotated and it is possible, therefore, to extract features that correlate with the extent of conservation of each structural type. An analysis of the nature of such USBs shows that additional lengths can either occur as extensions or insert in the middle of a protein structure. A class-specific trend for the type of structure adopted in indel regions has also emerged in the current analysis and each class prefers a specific type of structure (Figure 3, Figure S1 (b-d)). Figure 4 shows examples of different superfamilies that exhibit class-specific nature in accommodating length variations.

We find that in all superfamilies examined, the structurally unconserved regions amongst related proteins do not all retain a uniform pattern in solvent accessibility. This coincides with the expectation that it is in such regions that variation in lengths between proteins is introduced. To preserve the core scaffold, which may be the driving force in limiting the number of folds, indel regions are more prone to structural changes and this may result in greater solvent exposure in some proteins or alter protein surfaces to modify interaction interfaces. β-strands show a universal preference for solvent avoidance and this reflects the preference of such strands to avoid isolations from the protein core and integrate into the structure as well-ordered sheets (Table S2). In proteins of the α-β class, coils show a clear preference for solvent exposure, more so in α + β class superfamilies where they are vital in segregating α and β units. Inferences on solvent exposure, in the present analysis, are limited to individual domains of the proteins and do not consider multi-domain contexts and oligomerisation states of the proteins.

Based on the extent of length variation observed in different superfamilies, we have clustered all the superfamilies into length-rigid and length-deviant groups. Interestingly, length-rigid proteins are not as well-populated (as reflected in the number of members that are functionally diverse and in the number of families) as length-deviant proteins. While on the one hand, this does indicate that with the availability of more structures, trends in length-deviations could be affected in the identified rigid superfamilies, one may argue that such superfamilies are not preferred due to their strict length limitations and limited functional promiscuity (as reflected in the number of families). Length-deviant proteins, on the other hand, are found to include superfolds such as the P-loop NTP hydrolases, Ferredoxin folds etc., that have already been shown to be well represented in many genomes.

In many length-deviant protein superfamilies, despite large differences in length (over two fold in some cases), the core is often well preserved. The large additional lengths often do not involve the active site and in many cases they affect the oligomerization states and interacting surfaces of the protein (Ferritin like domain superfamily), introduce substrate-specificity (SH3 domains) and in some cases play an auto-regulatory role (Table S2). Since our analysis is derived from the PASS2 database of domain superfamilies, which in turn is guided by the domain definitions of SCOP, it is highly likely that severe length deviation, exhibited as additional domains, have escaped our attention.

These interesting trends that we have obtained on the nature and type of indels in protein superfamilies from different classes could impact the area of comparative modeling in indel regions of newer superfamily members. We have obtained some distinct trends on indels that are class-specific, with information on typical lengths. Such information, we expect, will be useful in the choice of specific structural types for newer relatives of protein superfamilies. Each superfamily shows a distinct trend in length variability and such information can be fed, by the assignment of variable gap penalties, into sequence alignment approaches to improve homology detection amongst members that vary considerably in length. We trust that such analyses would provide guiding principles during sequence searches, alignment and homology modeling of distant relationships.

## Authors' contributions

SS coded the algorithm, performed the analysis on the PASS2 dataset and drafted the manuscript. BP performed an initial manual analysis on five PASS2 superfamilies, MKG coded the JAVA based graphical viewer, Structview. NS and BO participated in design and review of the manuscript. RS conceived of the study, design, co-ordination and critically reviewed the manuscript. All authors read and approved the final manuscript.

## Supplementary Material

Additional file 1 CUSP: an algorithm to distinguish structurally conserved and unconserved protein domain alignments and its application in the study of large length variations. The data provided represent the various analysis carried out to determine and describe the length variation in the dataset (Section I: S1–S5) and also contains an example of the functional implications of indels in Cytochrome C domain superfamily (Section II). Additional figures and tables that support the data in the main text are also included. Click here for file

## References

[B1] Berman HM, Bhat TN, Bourne PE, Feng Z, Gilliland G, Weissig H, Westbrook J (2000). The Protein Data Bank and the challenge of structural genomics. Nat Struct Biol.

[B2] Wolf Y, Madej T, Babenko V, Shoemaker B, Panchenko AR (2007). Long-term trends in evolution of indels in protein sequences. BMC Evol Biol.

[B3] Zhang J (2000). Protein-length distributions for the three domains of life. Trends Genet.

[B4] Bhaduri A, Pugalenthi G, Sowdhamini R (2004). PASS2: an automated database of protein alignments organised as structural superfamilies. BMC Bioinformatics.

[B5] Pascarella S, Argos P (1992). Analysis of insertions/deletions in protein structures. J Mol Biol.

[B6] Reeves GA, Dallman TJ, Redfern OC, Akpor A, Orengo CA (2006). Structural diversity of domain superfamilies in the CATH database. J Mol Biol.

[B7] Chakrabarti S, Sowdhamini R (2004). Regions of minimal structural variation among members of protein domain superfamilies: application to remote homology detection and modelling using distant relationships. FEBS Lett.

[B8] Russell RB, Barton GJ (1994). Structural features can be unconserved in proteins with similar folds. An analysis of side-chain to side-chain contacts secondary structure and accessibility. J Mol Biol.

[B9] Kleywegt GJJT (1994). A super position. CCP4/ESF-EACBM Newsletter on Protein Crystallography.

[B10] Sali A, Blundell TL (1990). Definition of general topological equivalence in protein structures. A procedure involving comparison of properties and relationships through simulated annealing and dynamic programming. J Mol Biol.

[B11] Pugalenthi G, Bhaduri A, Sowdhamini R (2005). GenDiS: Genomic Distribution of protein structural domain Superfamilies. Nucleic Acids Res.

[B12] Murzin AG, Brenner SE, Hubbard T, Chothia C (1995). SCOP: a structural classification of proteins database for the investigation of sequences and structures. J Mol Biol.

[B13] Kabsch W, Sander C (1983). Dictionary of protein secondary structure: pattern recognition of hydrogen-bonded and geometrical features. Biopolymers.

[B14] Lee B, Richards FM (1971). The interpretation of protein structures: estimation of static accessibility. J Mol Biol.

[B15] Mizuguchi K, Deane CM, Blundell TL, Johnson MS, Overington JP (1998). JOY: protein sequence-structure representation and analysis. Bioinformatics.

[B16] Godzik A (1996). The structural alignment between two proteins: is there a unique answer?. Protein Sci.

[B17] Shindyalov IN, Bourne PE (1998). Protein structure alignment by incremental combinatorial extension (CE) of the optimal path. Protein Eng.

[B18] Marchler-Bauer A, Bryant SH (2004). CD-Search: protein domain annotations on the fly. Nucleic Acids Res.

[B19] Bashford D, Chothia C, Lesk AM (1987). Determinants of a protein fold. Unique features of the globin amino acid sequences. J Mol Biol.

[B20] Lecomte JT, Vuletich DA, Lesk AM (2005). Structural divergence and distant relationships in proteins: evolution of the globins. Curr Opin Struct Biol.

[B21] Nordlund P, Eklund H (1995). Di-iron-carboxylate proteins. Curr Opin Struct Biol.

[B22] Lougheed JC, Holton JM, Alber T, Bazan JF, Handel TM (2001). Structure of melanoma inhibitory activity protein, a member of a recently identified family of secreted proteins. Proc Natl Acad Sci USA.

[B23] Mizuguchi K, Go N (1995). Seeking significance in three-dimensional protein structure comparisons. Curr Opin Struct Biol.

[B24] Holm L, Sander C (1995). Dali: a network tool for protein structure comparison. Trends Biochem Sci.

[B25] Gibrat JF, Madej T, Bryant SH (1996). Surprising similarities in structure comparison. Curr Opin Struct Biol.

[B26] Orengo CA, Taylor WR (1996). SSAP: sequential structure alignment program for protein structure comparison. Methods Enzymol.

[B27] Johnson MS, Overington JP, Blundell TL (1993). Alignment and searching for common protein folds using a data bank of structural templates. J Mol Biol.

