# Supplementary material for: CUSP: an algorithm to distinguish structurally conserved and unconserved regions in protein domain alignments and its application in the study of large length variations
Source: BMC Struct Biol. 2008 May 31;8:28. doi: 10.1186/1472-6807-8-28 (PMC2423364; doi:10.1186/1472-6807-8-28)
Supplement: Additional file 1 — CUSP: an algorithm to distinguish structurally conserved and unconserved protein domain alignments and its application in the study of large length variations. The data provided represent the various analysis carried out to determine and describe the length variation in the dataset (Section I: S1–S5) and also contains an example of the functional implications of indels in Cytochrome C domain superfamily (Section II). Additional figures and tables that support the data in the main text are also included. [file 1472-6807-8-28-S1.pdf]

Additional information 1:

CUSP: an algorithm to distinguish structurally conserved and unconserved protein domain alignments and its application in the study of large length variations

Sankaran Sandhya [1], Barah Pankaj[1,2], Madabosse Kande Govind[1],
Bernard Offmann[3], Narayanaswamy Srinivasan[4] and Ramanathan Sowdhamini[1§]

I)Methods

S1. Percentage variability in structural types

Differences in the number of H, E or C between the longest and shortest member were also calculated for every superfamily:

$$\text{Variability in structural type} = \frac{(\text{total H/E/C in longest} - \text{total H/E/C in smallest})}{(\text{total H/E/C in longest})} * 100$$

S2. Extent of length variation accommodated in conserved and unconserved structural blocks

CUSP dissects structurally conserved blocks (SSB) from indels (USB). The length variation within each SSB and USB is determined by calculating the extent of length variation within each block as described in methods.

S3. Analysis of indel regions

Structurally unconserved regions [USB] of superfamilies were pooled together in a class-specific manner to determine their lengths and structural types. In addition, trends in these properties were also examined for the top 64 length deviant domain superfamilies as well as in the highly populated length deviant domain superfamilies. The impact of such indel regions and additional structural elements on protein function and structure was studied by examining the functional role of such additional structures in the most length-rigid and length-deviant superfamilies of our dataset and is discussed briefly here and in more detail elsewhere.

S4. Graphical representation of secondary structural alignments

Structview, a JAVA based stand-alone application, was developed for visual comparison of protein secondary structural alignments. Structview provides a 2-D visualization of secondary

structures in an alignment to enable a quick visual assessment of equivalent structures. The sequence and structural alignments displayed in separate panels, allows users to define color schemes for core secondary structural elements. The application calculates the number of protein secondary structures in each sequence and projects results in a tabular format to facilitate comparisons of the distribution of secondary structures across and within multiple families.

S5. Conservation of Solvent accessibility in conserved structural units

As described for the calculation of block scores in the CUSP algorithm (in methods), PSA scores were assigned to structural blocks to correlate conservation of solvent accessibility in structural blocks. Averaged PSA scores of each block were clustered into bins of 0-30%, 30-50% and >50% to indicate buried, partially exposed and exposed regions, respectively. The distribution of PSA scores in the 'high' conserved blocks of the three structural types [α, β and coil] were plotted to determine if solvent accessibility is conserved in a class-specific manner. Considerations of the PSA scores are limited to the treatment of the domains as monomers and multimeric assemblies are not included in the calculations.

II) Results

Functional role of indels in classicial domain superfamilies

Cytochrome C

The cytochrome-C superfamily includes many proteins that are vital components of electron transfer mechanisms in both prokaryotes and eukaryotes. Diverse sequences (~24% sequence identity) specify a compact cytochrome-C structure shared by all members. The Cytochrome C fold typically, consists of at least four α- helices that envelope a heme group, a short 3_{10}-helix and several turns. Related members show up to two-fold variation in length and are represented by 'dwarf' domains such as cytochrome C-551 and cytochrome C-553 [~70- 80 residues] as well as 'giant' domains such as methylamine dehydrogenase and cytochrome C-552 [~130-150 residues]. The CUSP algorithm when applied to alignments involving members of diverse lengths arrives at a structural consensus that detects the structural integrity of the heme-binding

pocket involving at least four α- helices and a predominantly hydrophobic pocket that is well conserved amongst all members[S1] The CXXCH motif that lies on spatial motifs originating from different structural elements is also detected. Alignments of the family involving different members and independently derived through CE[S2] show that the CUSP algorithm detects ~69% of the structurally equivalent residues detected by CE (Table S3). We have examined the functional roles of the additional structural motifs that appear in the giant members of the superfamily and find that they appear to characterize each protein and confer thermal stability to certain members. Most differences in length are due to variations in the lengths of surface loops connecting the α- helices.

Supplementary Figures

Figure S1:

a) Extent of length variation accommodated in CUSP-delineated SSB and USB across domain superfamilies from all classes.

b) Extent of length variation amongst the domain members of the 64 length deviant domain superfamilies (1-64 on the X axis correspond to the 64 length domain superfamilies listed in Table 2).

c) Distribution of structural types in indel regions of the 64 length deviant domain superfamilies (1-64 on the X axis correspond to the 64 length domain superfamilies listed in Table 2).

d) Structural type in indel regions of the highly populated domain superfamilies listed in Table 1.

Figure S2:

a) Distribution of average PSA scores in SSB [α-helix, β- strand, coils] and USB for 81 superfamilies in the β-class.

b) Distribution of PSA scores in 'high conserved' structural blocks [SSB] in the four classes.

Figure S3: PSA distribution in SSB and USB regions of protein superfamilies from alpha class.

Figure S4: PSA distribution in SSB and USB regions of protein superfamilies from alpha/beta class.

Figure S5: PSA distribution in SSB and USB regions of protein superfamilies from alpha +beta class.

Additional tables:

Table S1: List of 'Length-rigid superfamilies' (>4 members) across all the structural classes.

Table S2:List of ‘Length-deviant superfamilies’ (>4 members) across all the structural classes and structural and functional implications of additional lengths.

Table S3: Comparison of structurally conserved residue types (H, C and E) reported by CUSP, CE and CDD

Table S4: Differences in number of secondary structures [Helix, Strand and Coil: H,E,C] between longest and shortest members of ‘length-rigid’ superfamilies.

Table S5: Differences in number of secondary structures [Helix, Strand and Coil: H,E,C] between longest and shortest members of ten ‘length-deviant’ superfamilies.

References

S1. Benini S, Gonzalez A, Rypniewski WR, Wilson KS, Van Beeumen JJ, Ciurli S: **Crystal structure of oxidized Bacillus pasteurii cytochrome c553 at 0.97-A resolution**. *Biochemistry* 2000, **39**(43):13115-13126.

S2. Shindyalov IN, Bourne PE: **Protein structure alignment by incremental combinatorial extension (CE) of the optimal path**. *Protein Eng* 1998, **11**(9):739-747.

S1

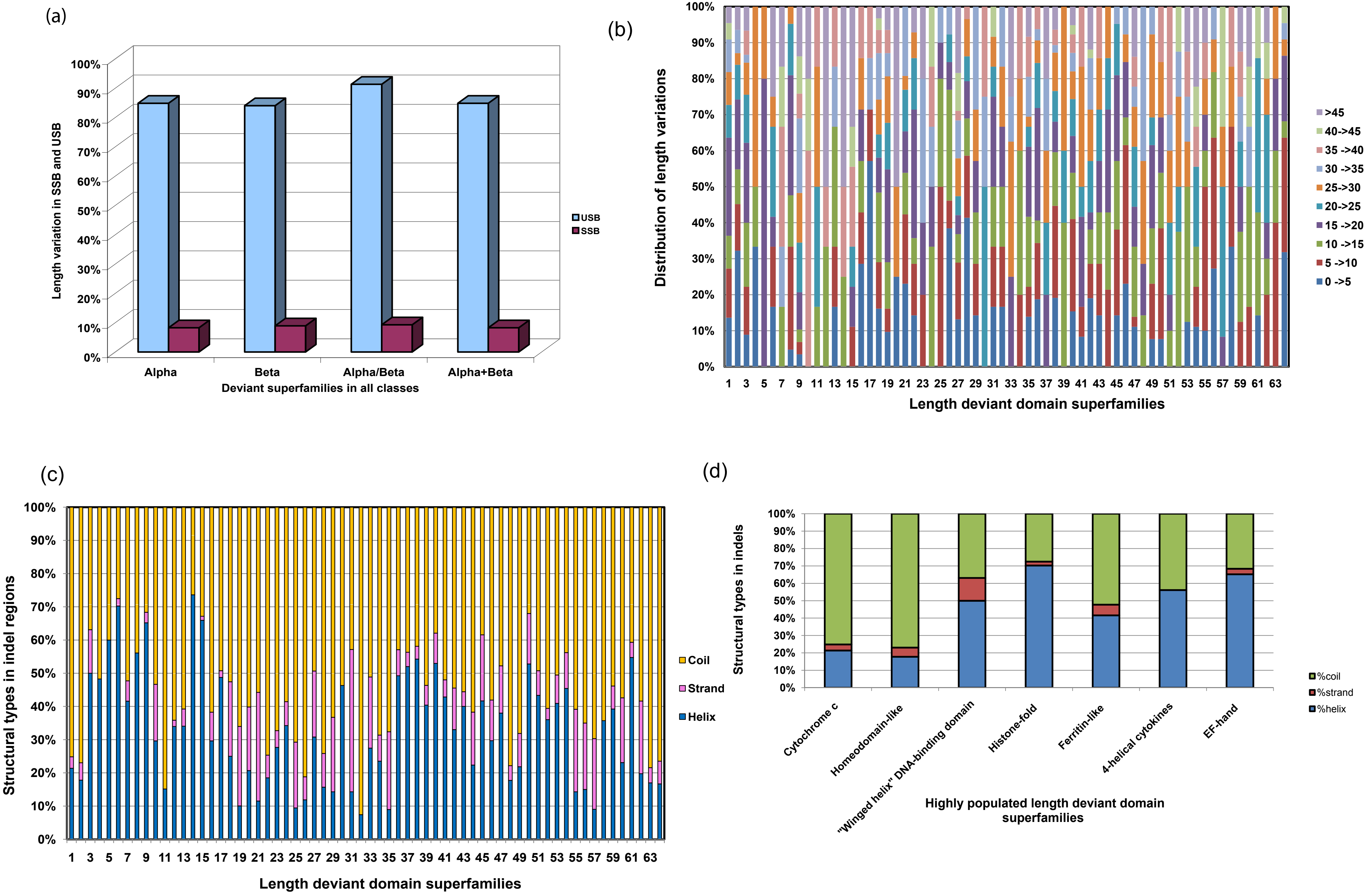
(a)
100%
90%
80%
70%
60%
50%
40%
30%
20%
10%
0%
Length variation in SSB and USB
USB
SSB
Alpha
Beta
Alpha/Beta
Alpha+Beta
Deviant superfamilies in all classes
(b)
Distribution of length variations
>45
40->45
35 ->40
30 ->35
25->30
20->25
15 ->20
10 ->15
5 ->10
0 ->5
1 3 5 7 9 11 13 15 17 19 21 23 25 27 29 31 33 35 37 39 41 43 45 47 49 51 53 55 57 59 61 63
Length deviant domain superfamilies
(c)
Structural types in indel regions
Coil
Strand
Helix
1 3 5 7 9 11 13 15 17 19 21 23 25 27 29 31 33 35 37 39 41 43 45 47 49 51 53 55 57 59 61 63
Length deviant domain superfamilies
(d)
Structural types in indels
%coil
%strand
%helix
Cytochrome c
Homeodomain-like
"Winged helix" DNA-binding domain
Histone-fold
Ferritin-like
4-helical cytokines
EF-hand
Highly populated length deviant domain superfamilies

S2

(a)

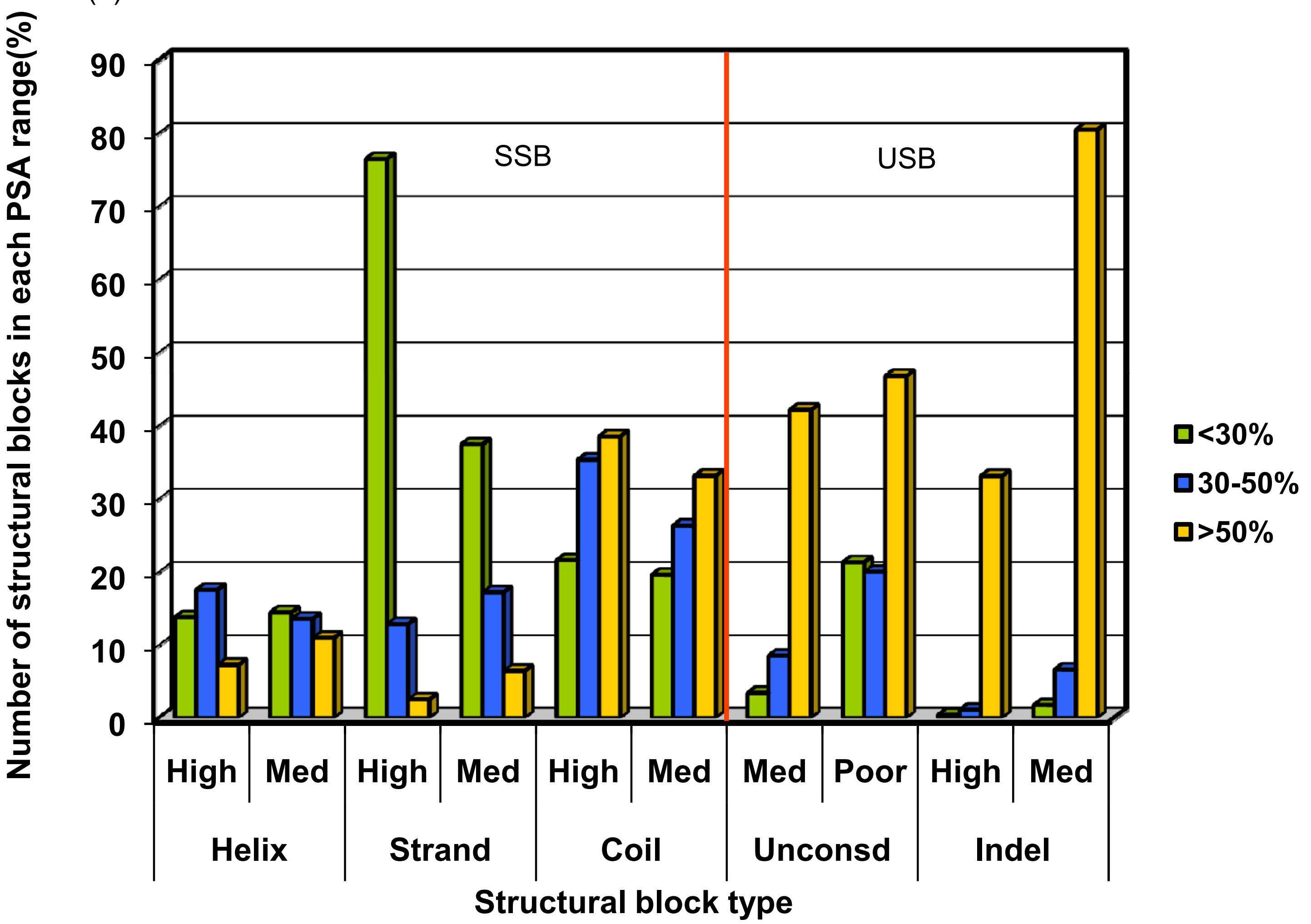
Number of structural blocks in each PSA range(%)
90
80
70
60
50
40
30
20
10
0
SSB
USB
High
Med
High
Med
High
Med
Med
Poor
High
Med
Helix
Strand
Coil
Unconsd
Indel
Structural block type
<30%
30-50%
>50%

(b)

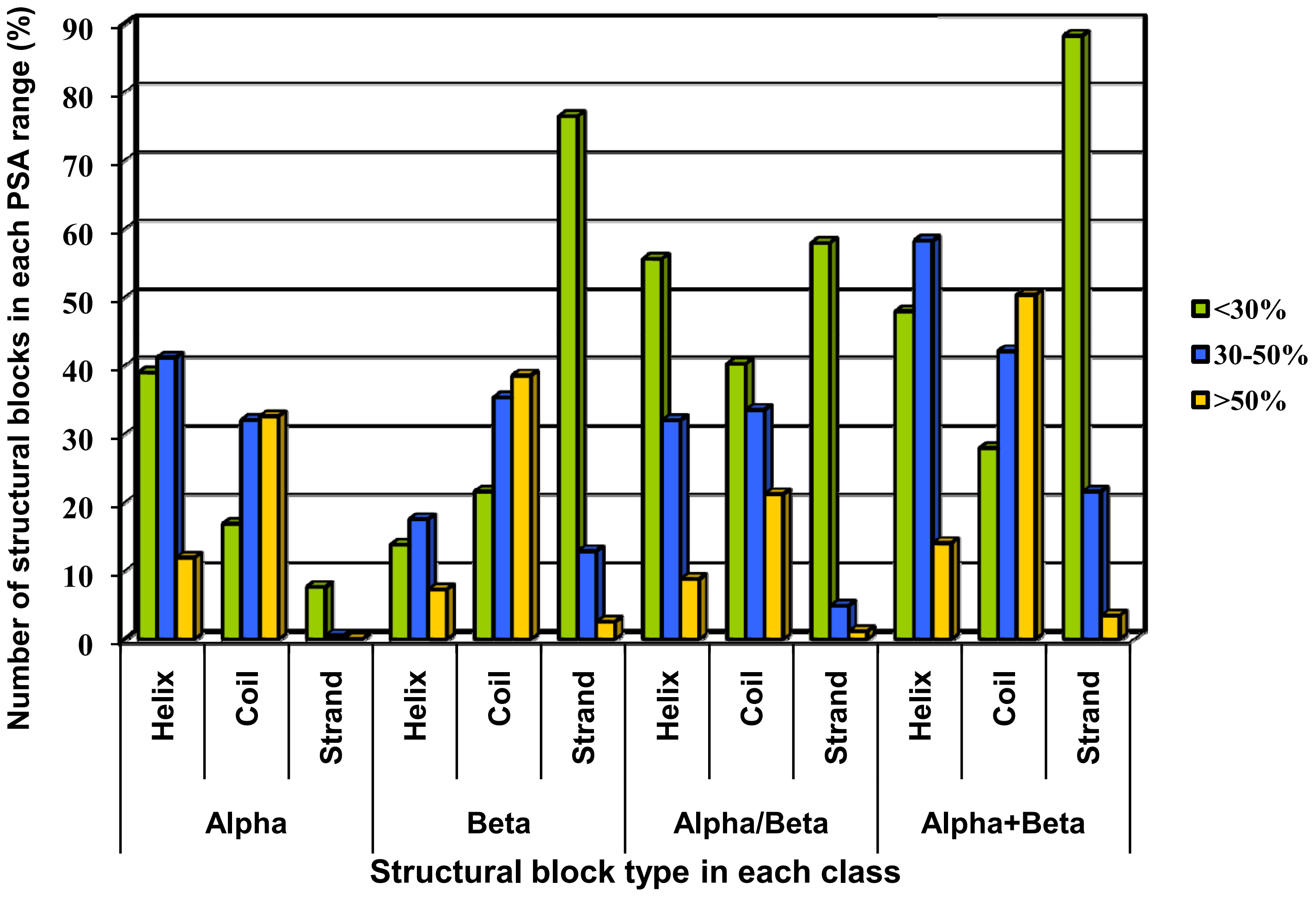
Number of structural blocks in each PSA range (%)
90
80
70
60
50
40
30
20
10
0
Helix
Coil
Strand
Helix
Coil
Strand
Helix
Coil
Strand
Helix
Coil
Strand
Alpha
Beta
Alpha/Beta
Alpha+Beta
Structural block type in each class
<30%
30-50%
>50%

S3

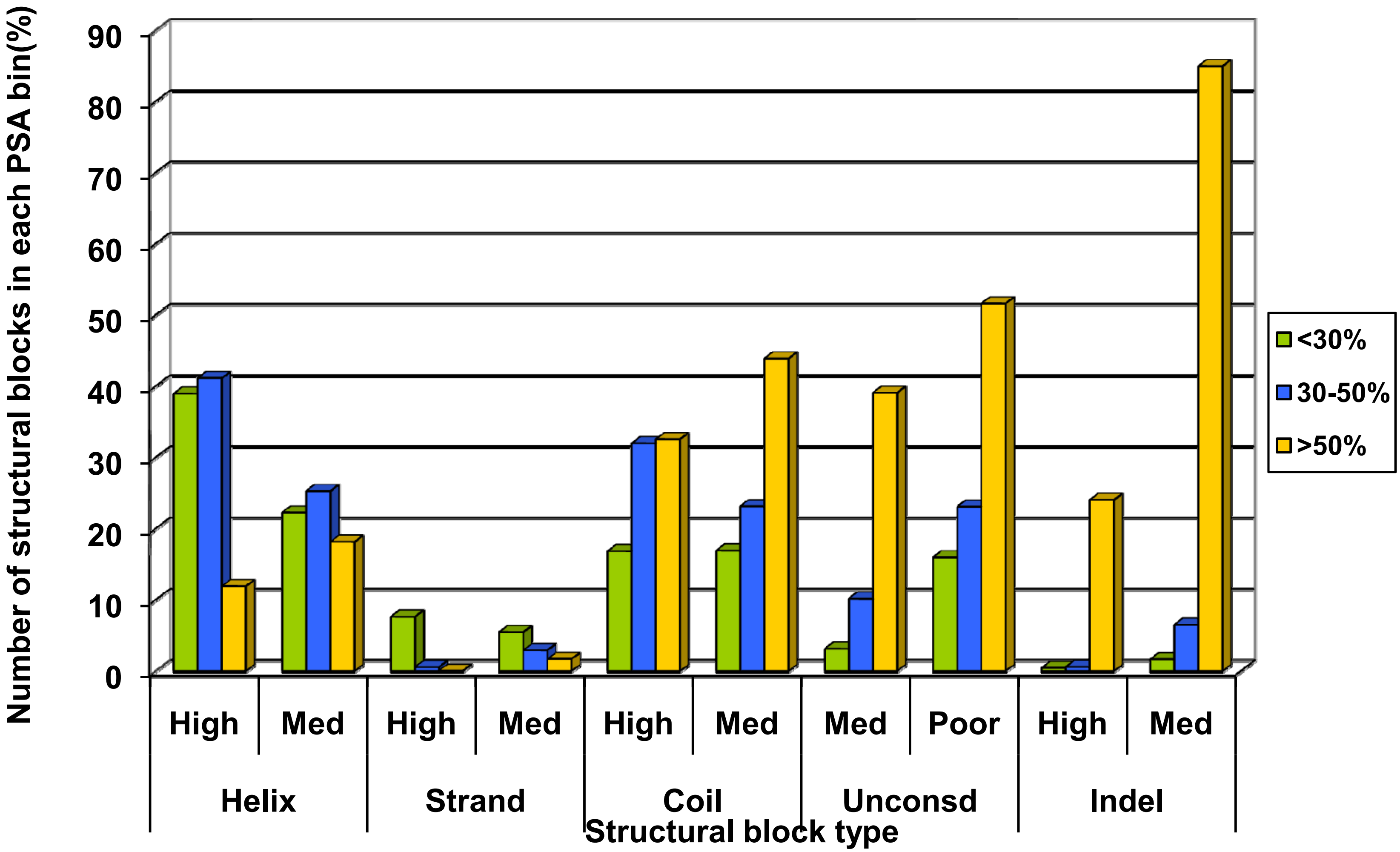

Number of structural blocks in each PSA bin(%)
90
80
70
60
50
40
30
20
10
0
<30%
30-50%
>50%
High
Med
High
Med
High
Med
Med
Poor
High
Med
Helix
Strand
Coil
Unconsd
Indel
Structural block type

S4

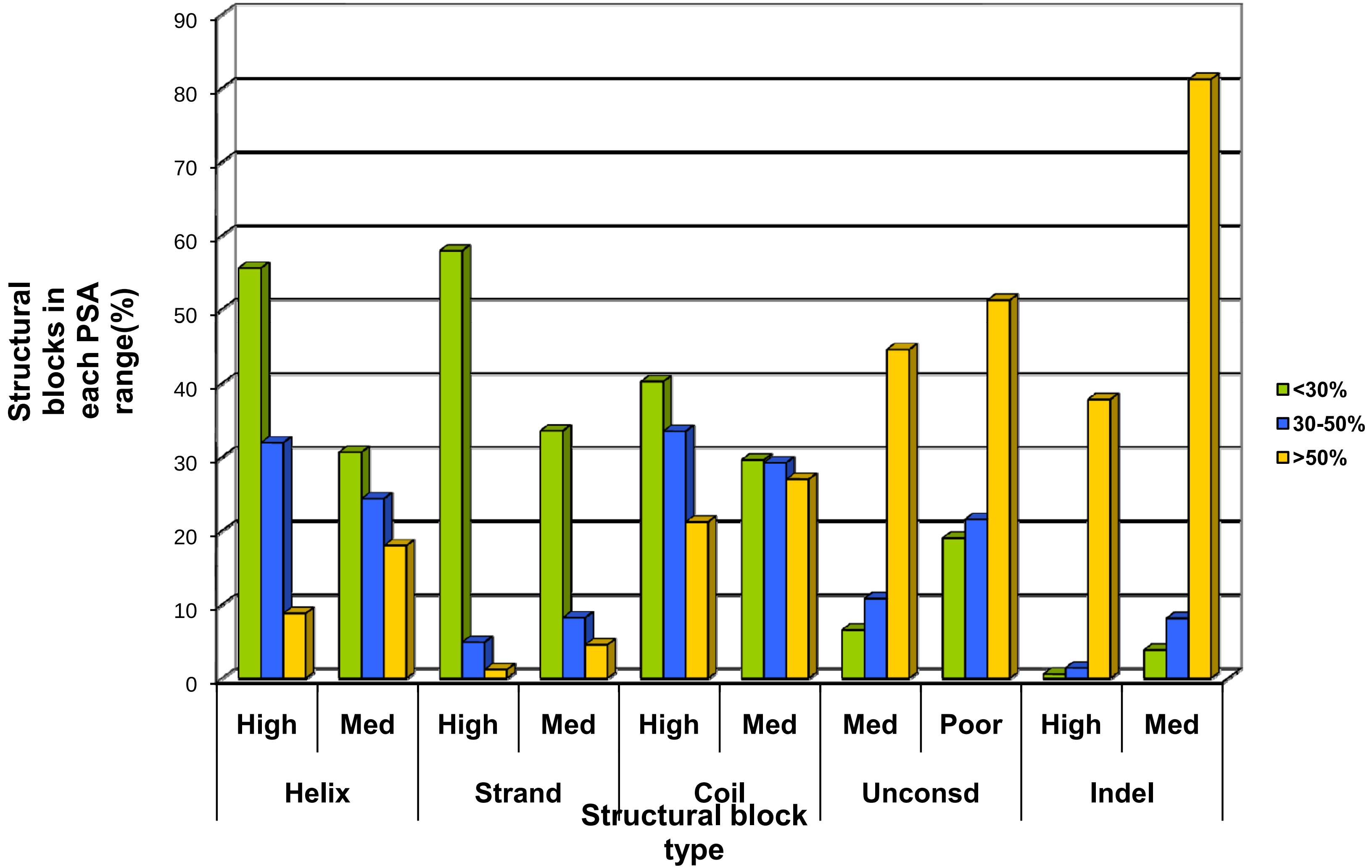
90
80
70
60
50
40
30
20
10
0
Structural blocks in each PSA range(%)
<30%
30-50%
>50%
High
Med
High
Med
High
Med
Med
Poor
High
Med
Helix
Strand
Coil
Unconsd
Indel
Structural block type

S5

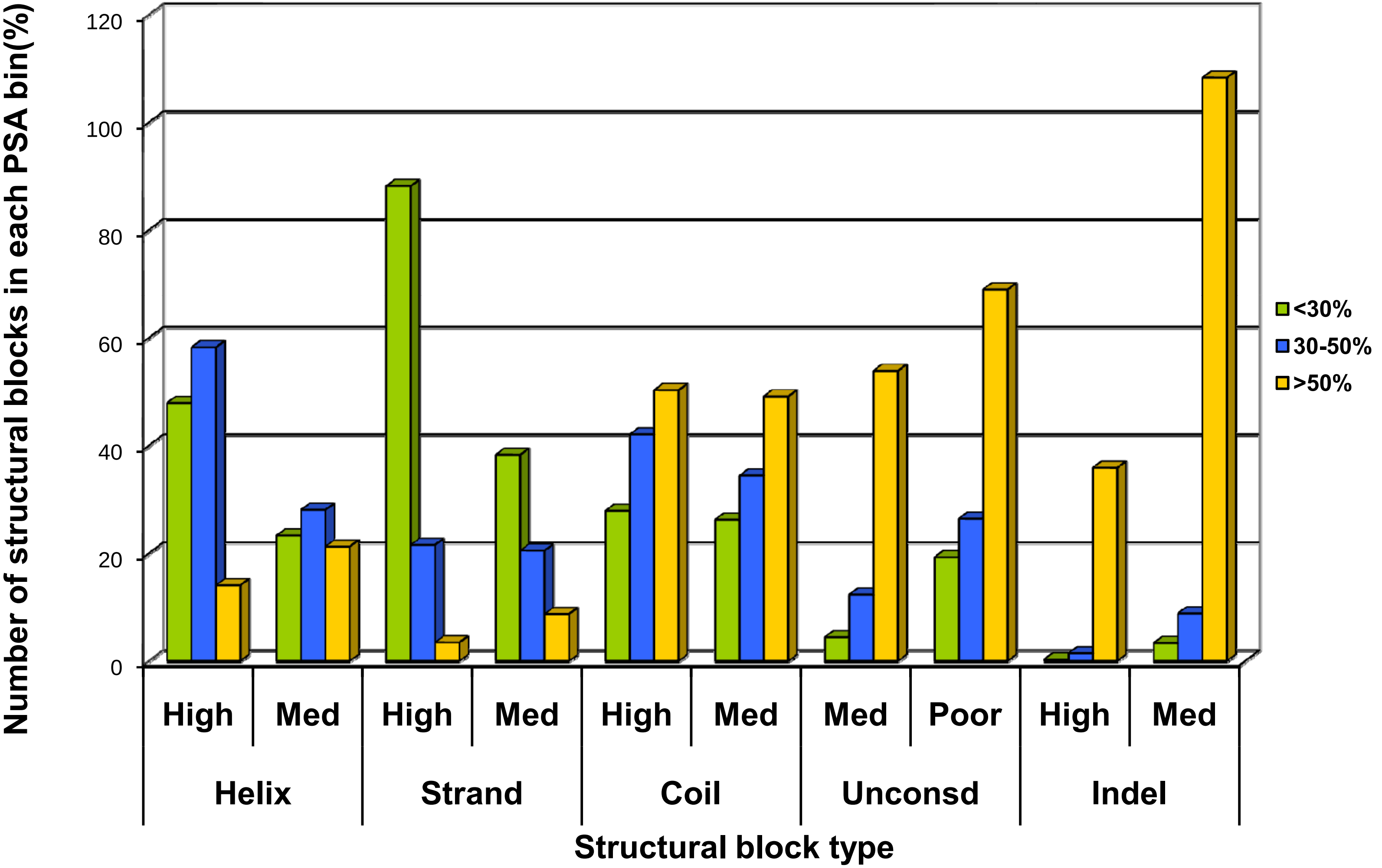

Number of structural blocks in each PSA bin(%)
120
100
80
60
40
20
0
<30%
30-50%
>50%
High
Med
High
Med
High
Med
Med
Poor
High
Med
Helix
Strand
Coil
Unconsd
Indel
Structural block type

Table S1: List of 'length-rigid superfamilies' (>4 members) across all the structural classes.

**S.No**	**Scop class**	**No of members**	**Average domain size**	**Sequence Identity**	**Description**
1	α	8	417	21	Cytochrome P450
2	α	6	323	14	Terpenoid synthases
3	α	8	250	25	Nuclear receptor ligand-binding domain
4	α	5	204	23	DNA-glycosylase
5	α	5	114	26	Calponin-homology domain, CH-domain
6	β	7	145	26	TNF-like
7	β	5	135	29	cAMP-binding domain like
8	β	10	133	24	C2 domain (Calcium/lipid domain, CaLB)
9	β	5	118	22	Actin-crosslinking proteins
10	β	6	94	29	Invasin/intimin cell-adhesion fragments
11	β	5	75	33	Sm-like ribonucleoproteins
12	α/β	6	474	23	ALDH-like
13	α/β	8	299	15	Zn-dependent exopeptidase
14	α/β	6	254	23	Purine and uridine phosphorylases
15	α + β	6	239	22	Metallo-hydrolase/oxidoreductase
16	α + β	7	253	30	Ribosome inactivating proteins (RIP)
17	α + β	11	167	30	Lactate & malate dehydrogenases, C-terminal domain
18	α + β	7	111	36	Superantigen toxins, C-terminal domain
19	α + β	7	151	36	UBC-like
20	α + β	12	124	22	DNA clamp
21	α + β	17	87	29	RNA-binding domain, RBD
22	α + β	8	70	36	Metal-binding domain
23	α + β	10	70	38	Interleukin 8-like chemokine
24	α + β	5	67	33	Chromo domain like

Table S2:List of length deviant domain superfamilies, structural and functional implications of additional lengths								
**S.No**	**Class**	**Description**	**Scop code**	**No_members**	**Average domain size**	**Sequence identity (%)**	**Domain size in Giant and dwarf domain**	**Structural/Functional role**
1	α	Cytochrome c	46626	22	101	24	G:1iqca2 (158), D:1c75a (71)	**Thermal stability**: Two fold length occurs predominantly in additional helices and long loops that pack tightly against the domain and bury the cytochrome deep into the structure.
2	α	Homeodomain-like	46689	32	64	26	G:1igna2(103), D:1gdta1(43)	**Diverse functional repertoire:**DNA recognition domains that differ in the manner in which DNA is recognised. The dwarf domain recognises and binds to short DNA sites at which they cleave the DNA backbone, exchange the two DNA helices involved and rejoin the DNA strands.Giant domains have more diverse functional repertoire and binds telomeric DNA as well as involves in the activation and repression of transcription.
3	α	"Winged helix" DNA-binding domain	46785	48	88	21	G:2foka2(138), D:1j75a(57)	**Complex domain architecture** in the giant domain of FOk1 restriction endonuclease. It consists of an N-terminal DNA recognition domain and a C-terminal cleavage domain. The structure reveals a dimer, in which the dimerization interface is mediated by the C terminal domain.The recognition domain is comprised of three smaller subdomains (D1, D2, and D3) that are evolutionarily related to the helix-turn-helix- containing DNA-binding domain. The winged helix domain has been embellished extensively in D1 and D2, whereas in D3 it has been co-opted for protein-protein interactions.

4	α	C-terminal effector domain of bipartite response regulator	46894	6	92	32	G:1fc3a(119), D:1fsea-(67)	**Additional structural elements:** The C terminal catalytic domain of giant member possesses additional secondary structures such as an N-terminal helix. The core scaffold that interacts with DNA is well conserved across all the members and the exact functions of the additional lengths yet to be resolved.
5	α	Putative DNA-binding domain	46955	5	90	27	G:1exja1(118), D:1jjcb2(75)	**Domain combinations**: Domain members of this superfamily occur in diverse proteins and also differ in the number of copies of the structural domain
6	α	Histone-fold	47113	12	88	29	G:1f1ea-(147), D:1bh9a-(45)	**Number of copies vary in different proteins**: Nucleosome core histones contain 2 copies of four histones, while archael members possess only a single copy.
7	α	Ferritin-like	47240	12	259	17	G:1mtyd(512), D 1dvba1(147)	**Domain interactions**: Although giants and dwarf domains are di-iron carboxylate proteins and conserve interactions with the Fe strictly, giant domains are associated with newer interaction interfaces. The number of interacting domains in the giant member is more than ruberythrin. This difference could account for the acquisition of extra structural elements that can interact with different domains.
8	α	4-helical cytokines	47266	22	142	18	G:1lki--(172), D:1hu1a(108)	**Oligomer interface**: The dwarf domain is a functional dimer involving a close association of the 2 chains. Each domain is formed by participation of residues from both the chains and involve domain swapping events to form a functional module.
9	α	EF-hand	47473	35	125	23	G:1el4a-(194), D:1ctda-(34)	**Functional repertoire and variations in repeat copies**: A conserved structural scaffold that occurs in diverse proteins. Members differ in the number of EF hand repeats

10	α	Met repressor-like	47598	5	75	33	G:1mnta-(132), D:2cpga-(43)	**Oligomer interface**: Dwarf domain serves as a prototype of the domain family. Giant domain member, a functional tetramer resembles the dwarf domain at its N-terminal. The C terminal end of the giant is long and acquires additional secondary structures that are involved in the formation of a tetramerisation domain
11	α	IHF-like DNA-binding proteins	47729	6	76	37	G:1exea-(99), D:1hns--(47)	**Thermal stability:** Tighter packing at the dimer interface and the involvement of additional structures in creating an additional DNA binding interface
12	α	6-phosphogluconate dehydrogenase C-terminal domain-like	48179	6	191	22	G:1pgja1 (297), D:1dlja1(98)	**Dimer formation**: Additional length involved in dimer interface in the giant domain. The dwarf domain is truncated and sandwiched between an N and C terminal domain belonging to different superfamilies although a fair amount of structural similarity exists between the N and C terminal domain.
13	α	Terpenoid cylases/Protein prenyltransferases	48239	6	308	18	G:1d8db-(407), D:5eau-1(200)	**Substrate recognition**: Protein superfamilies recognises diverse substrates and additional structures in the different members facilitate such recognition
14	α	ARM repeat	48371	9	369	17	G:1qbkb-(856), D:1bpoa1(157)	**Structural repeat and interaction interface**: Members differ in number of repeating domain copies. Presentation of new interaction interfaces
15	α	TPR-like	48452	9	202	21	G:1hz4a-(366), D:1hxia-(108)	**Structural repeat**: Members differ in number of repeating domain copies. Presentation of new interaction interfaces
16	β	Carbohydrate-binding domain	49384	7	136	23	G:1qba-2(173), D:1e5ba-(87)	**Domain interactions**: Giant member has multiple domains and the additional structures in the CBD domain are involved in these additional domain -domain interactions
17	β	p53-like transcription factors	49417	7	184	18	G:1bg1a2(254), D:1h9da-(125)	**Substrate recognition**: Giant domains respond to a variety of cytokines and growth factors and differ in the nature of the interacting domain partners from the dwarf domain

18	β	Cupredoxins	49503	32	146	19	G:1aoza3(209), D:2cbp--(96)	**Domain organisation and functional type**: Multicopper blue proteins (MCBPs) are multidomain proteins that utilize the distinctive redox ability of copper ions. There are a variety of MCBPs that have been roughly classified into three different groups, based on their domain organization and functions: (i) nitrite reductase-type with two domains, (ii) laccase-type with three domains, and (iii) ceruloplasmin-type with six domains.
19	β	Viral coat and capsid proteins	49611	31	227	14	G:1ihma-(492), D:1stma-:(141)	**Interaction interfaces that dictate function**: In the giant member, the capsid protein has a protruding (P) domain connected by a flexible hinge to a shell (S) domain that has a classical eight-stranded beta-sandwich motif. The structure of the P domain is unlike that of any other viral protein with a subdomain exhibiting a fold similar to that of the second domain in the eukaryotic translation elongation factor-Tu. This subdomain, located at the exterior of the capsid, has the largest sequence variation among Norwalk-like human caliciviruses and is likely to contain the determinants of strain specificity and cell binding
20	β	Viral proteins	49749	4	313	25	G:1p30a1(534), D:1hx6a2(140)	**Protein stability and size**: Viral jelly roll, characteristic of this superfamily interact with varying lengths of interconnecting loops. These loops are involved in different subunit interactions.
21	β	Concanavalin A-like lectins/glucanases	49899	26	197	14	G:1dyp ,D:1slt (133)	**Quarternary interactions:** Carbohydrate recognition is mediated by loops of variable length in different members.
22	β	SH3-domain	50044	14	71	33	G:1i1j(106), 1gcq (56)	**New interaction interface**: Additional residues involved in interactions involving other domains.

23	β	Translation proteins SH3-like domain	50104	5	100	27	G:1jj2a1(147), D:1rl2a1(69)	**Interaction interfaces**:1jj2 is a multi chain protein involved in extensive interactions. It has several chains each specifying an entirely different domain or many different domains. 3 chains specify the parent domain superfamily. This multi chain occurrence may satisy its functional role since it's a ribosomal protein involving many interacting partners. this domain whether single or multiple exists with multiple other domains
24	β	GroES-like	50129	6	166	26	G:1heta1(224), D:1jh2a-(99)	**Oligomer formation**: Both giant and dwarf domains differ in their final quarternary assemblies and additional lengths involve in these diverse interactions
25	β	PDZ domain-like	50156	10	99	31	G:1il6--(130), D:1kwaa(88)	**Substrate recognition**: Loops of diverse lengths lie near the PDZ-like binding site and alter conventional binding properties so that giant domains like interleukin differ in location and in nature of recognised substrate.
26	β	Bacterial enterotoxins	50203	13	99	23	G:3seb1(121), D:1c4qa- (69)	**New interaction interfaces**: Typically a 2 domain protein with an N terminal OB fold and a C terminal B grasp fold. Length variations in the giant member of this superfamily occur as longer loops between connecting strands of the N terminal OB fold domain. These loops are involved in modifications to the conventional T cell receptor binding site that can affect the potency of these superantigen toxins
27	β	Nucleic acid-binding proteins	50249	39	112	20	G:1jb7b(216); D:1bkb2(62)	**Domain architecture**: Most proteins are multi domain proteins, either on single or multiple chain. Strong requirement to interact with several partnering domains.

28	β	Trypsin-like serine proteases	50494	30	225	24	G:1dlea(288), D:2hrva(139)	**New domain interactions, cofactor and substrate binding:** Giant domains possess unique bulky and rigid motifs on the back, three distinct deletions on the right and six loop insertions around the active site. Given that the giant members are multidomain SPs and require cofactor binding to express proteolytic activity fully, it seems possible that these unique regions could be involved in the domain–domain interactions, cofactor binding
29	β	ADC-like	50692	7	119	25	G:1eu1a1 (155), D:1cr5a1(82)	**Oligomer formation**: Proteins like Arsenite oxidase Rieske subunit show multiple chains, 4 chains harbour single domain copies of the ISP domain and 4 chains are multi-domain with one domain specifying the ADC domain like superfamily and the other domain usually DMSO reductase domain. Repeats of the domain on a single chain are not observed but domain duplication across multiple chains observed.
30	β	PK beta-barrel domain-like	50800	5	127	31	G:1jhda1(173), D:1e0ta1(98)	**Domain interfaces and domain linkers**: The architecture of PK consists of an assembly of domains and subunits in which allosteric and catalytic sites are able to communicate with each other across relatively long distances. Various protein regions, including domain interfaces and flexible domain linkers, couple changes in the tertiary and quaternary structures to alterations in the geometry of the active and allosteric sites.

31	β	alpha-Amylases, C-terminal beta-sheet domain	51011	12	78	26	G: (113), D:1avaa1(57)	**Additional sub-domain like features**:In the N-terminal region, isoamylase (giant domain) has a novel extra domain that we call domain N, whose three-dimensional structure has not so far been reported. It has a (beta/alpha)8-barrel-type supersecondary structure in the catalytic domain common to the alpha-amylase family enzymes, though the barrel is incomplete, with a deletion of an alpha-helix between the fifth and sixth beta-strands.
32	β	WW domain	51045	6	38	48	G:1i5hw-(50), D: 1e0na-(27)	**Domain combinations**: Small domain modules that recognise Pro-rich sequences. Some modules have evolved alternate modes of action
33	β	RmlC-like cupins	51182	8	243	17	G:1pmi (439),D:1dgw (177)	**Higher order complexes:** Cupin superfamily exists in diverse quarternary arrangements and such requirements may be facilitated by length changes.
34	β	Rudiment single hybrid motif	51246	5	85	27	G:1dv1a1(116), D:1e2wa2(64)	**Interaction interfaces and differences in substrate**
35	β	E set domains	81296	42	122	19	G:1hc2-3(244), D:1i9wa1(77)	**Domain partnerships and interaction interfaces**
36	α/β	(Trans)glycosidases	51445	46	360	11	G:1byb--(490), 1jfxa-(217)	**Alterations to ligand binding sites**: Longer loops in giant domain alter the presentation of the active site to the substrate. 3 long loops occuring as indels line the active site of the giant domain
37	α/β	Phosphoenolpyruvate/pyruvate domain	51621	5	341	20	G:1dqua-(513), D:1e0ta2(231)	**Regulatory function and structural role**:In the phosphoenol pyruvate binding domain, giant members such as PEP carboxylase have acquired additional helices in the C-terminal that harbor an inhibitor binding site. Repeating copies of the domain seen in members that are domain swapped dimers

38	α/β	NAD(P)-binding Rossmann-fold domains	51735	49	183	16	G:1hwxa1(293), D:1euca1(130)	**Substrate specificity and oligomer interactions** vary between members. The giatn domain has additioanl antenna like elements protruding from the trimer that act as intersubunit conduits during regulation
39	α/β	Adenine nucleotide alpha hydrolases-like	52402	6	240	19	G:1ct9a1(305), D:1gpma1(175)	**Substrate diversity** and additional structural elements in the giant domain that modify surface properties of the giant domain.
40	α/β	P-loop containing nucleotide triphosphate hydrolase	52540	63	221	14	G:1g41a-(334), D:1a1va1 (135)	**Functional variety and topological differences**: A unifying element of the superfamily is the conservation of the P loop motif that serves as an Atp recognition module. Each domain member however shows a large diversity in substrate, location and domain organisation. Topological differences in connectivity of strands also result in over two fold length variations
41	α/β	(Phosphotyrosine protein) phosphatases II	52799	12	234	23	G:1lara1(317), D:1mkp--(144)	**Dimer formation**: Additional length involved in dimer interface in the giant domain which also occurs as a structural domain repeat.
42	α/β	Thioredoxin-like	52833	42	109	21	G:1prxa-(219), D:1g7oa2(75)	**Dimer formation**: The giant domain involves additional length in the formation of a dimerisation interface
43	α/β	Aminoacid dehydrogenase-like, N-terminal domain	53223	7	153	24	G:1hwxa2(208), D:1b0aa2(121)	**Oligomeric interface** and acquisition of additional functional features
44	α/β	PRTase-like	53271	14	194	18	G:1ecfa1(242),D:1dkra2 (149)	**Substrate recognition**: Loops of diverse lengths lie in subunit interfaces and involve in diverse roles such as catalysis, allostery. Short loops are seen in dimeric PRTases since they lie adjacent to active site of adjacent subunits. Longer loops are often observed in monomeric PRTases. In addition, hoods of variable lengths recognize distinct substrates and are involved in specific reactions

45	α/β	S-adenosyl-L-methionine-dependent methyltransferase	53335	21	238	14	G:1f3la-(320),D:1ej0a-(179)	**Function regulation and specificity**: In the giant domain, additional lengths form a b-rich subdomain containing residues that interact with substrate and introduce functional specificity. Each member methylates specific substrates. In addition, it is implicated in an autoregulatory role in the predicted biological dimer.
46	α/β	Nucleotide-diphospho-sugar transferases	53448	13	251	14	G:1fo8a-(330), D:1e5ka-(188)	**Domain interaction interfaces**: Giant domains oligomerise and indels involved in presentation of interaction interfaces with different domains.
47	α/β	alpha/beta-Hydrolases	53474	39	354	12	G: 1dx4a(537), D: 1fj2a(229)	**Oligomer formation and subunit assembly** differs across the diverse members. Range of substrates recognized also expansive.
48	α/β	"Helical backbone" metal receptor	53807	7	400	19	G:1mioa-(525), D:1efdn-262)	**Domain interaction interfaces**: Giant domains oligomerise and indels involved in presentation of interaction interfaces with different domains.
48	α/β	Periplasmic binding protein-like I	53822	13	376	16	G: 1ewka(448), D: 1byka (255)	-
50	α/β	Periplasmic binding protein-like II	53850	15	255	15	G:1cb6a2(357), D:1gv8a-(159)	**Structural repeats**:Tandem structural repeats of the domain in giant members
51	α/β	Thiolase-like	53901	12	193	19	G:1 afwa1(266), D:1afwa2(124)	**Dimer interface**: N terminal domain contrubutes additional residues for tight dimer interactions. Consists of two similar domains related by pseudo dyad
52	α + β	Ankyrin repeat	48403	8	176	26	G:1sw6a-(254), D:1myo--(118)	**Structural repeat** domain that varies in the number of repeats in different members.Structural repeats of beta(2)-alpha(2) motif dictate diverse domain sizes and form new interaction interfaces
53	α + β	Lysozyme-like	53955	9	187	19	G:1qus (321),D:1iiz (119)	**New interaction interface:** Additional residues may be involved in membrane interactions

54	$\alpha + \beta$	Cysteine proteinases	54001	9	278	23	G:3gcb--(458), D:1qmya-(156)	**Interaction interface and domain organisation**:Constituent family members are known to have many insertions into and circular permutation of the catalytic core. Some members have homologous domains on multiple chains such as the FMDV leader protease. Giant members such as the bleomycin hydrolase has more insertions into the common papain-like fold.
55	$\alpha + \beta$	Ribosomal protein S5 domain2 like	54211	11	134	20	G:1fi4a1(185), D:1pkp-1(71)	**Domain architecture**: Primarily multi domain in protein and found in association with diverse interacting partners. Multiple domains specified by single chain in many protein members. Tandem repeats of partner domains observed in many cases. Tandem duplications of parent domain observed in Polynucleotide phosphorylase, DNA gyrase B which is additionally also multi domain in nature
56	$\alpha + \beta$	FAD-linked reductases, C-terminal domain	54373	11	101	20		-
57	$\alpha + \beta$	MHC antigen-recognition domain	54452	13	143	25		-
58	$\alpha + \beta$	POZ domain	54695	6	95	29	G:1buoa-(121), D:1fs1b2 (61)	**Oligomer formation**: Single domain protein that is usually in a single chain. Elongins from human are complex proteins constituted by multiple chains, each chain specifies a single domain that belongs to diverse families. Cyclin A, the dwarf member is again a multi chain protein, more than one domain is specified in each chain while in the other multi chain protein members each chain specifies a single domai

59	α + β	4Fe-4S ferredoxins	54862	8	95	33	G:1h7w5a (173), D:1vjw--(59)	**Thermal stability:** Dwarf domain from a thermophile is an extremely rigid domain.These are primarily due to a stabilization of alpha helices, replacement of residues in strained conformation by glycines, strong docking of the N-terminal methionine and an overall increase in the number of hydrogen bonds. Most of these features stabilize several secondary structure elements and improve the overall rigidity of the polypeptide backbone.
60	α + β	Metalloproteases ("zincins"), catalytic domain	55486	6	233	21	G:1lml--(465), D:1c7ka-(132)	**Structural elements** alter surface proteins and contribute additional domainsGiant domain is a novel member of the domain superfamily and has additional regions that are nearly like two novel folds. Conserved properties of the zincins are retained in the N terminal domain that has additional residues bordering the active site. Additional residues contribute to alterations in surface properties of the protein
61	α + β	Tetrahydrobiopterin biosynthesis enzymes-like	55620	7	155	19	G:1a8ra-(221), D:1b91a-(119)	**Oligomer formation**:Known members form wide oligomeric barrels of diverse sizes
62	α + β	Acyl-CoA N-acyltransferases (Nat)	55729	10	194	18	G:1bob--(306), D:1bo4a-(137)	**Oligomer interface**: Giant domains are involved in higher order oligomer formation and new interaction interafaces.
63	α + β	Phospholipase D/ nuclease	56024	5	215	20	G:1f0ia1(257), D:1byra (149)	**Multiple repeats:** Giant members possess two copies of the domain that relate in a pseudo-dyad symmetry. Longer loops pack the two domains together. Some loops may involve in enzyme interactions with membrane. Dwarf domains are functional dimers and possess shorter loops.

64	$\alpha + \beta$	C-type lectin-like	56436	22	120	25	G:1koe--(172), D:1prea1(83)	**Oligomer interface**: Multiple copies of the domain specified in separate chains such as in snake coagglutinin alpha chain. In surfactant protein as well as Pertussis toxin, found in association with other domains in an oligomer.

Table S3: Comparison of structurally conserved residue types (H,C and E) between CUSP, CE and CDD

S.No	Superfamily	Scop code	Number of members			Av_domain size	Conserved residues			^CUSP performance *vis a vis*	
			PASS2	CE	CDD*		CUSP	CE	CDD	CE (in %)	CDD(in %)
1	4 helical cytokines	47266	22	13	2(6)	142	44	56	156	79	28
2	Concanvalin	49899	26	9	3(10)	197	43	115	184	37	23
3	PEP domain	51621	5	4	2(8)	341	280	238	396	100	71
4	Phospholipase D	56024	5	3	3(10)	215	168	139	123	100	100
5	Cytochrome C	46626	22	10	4(10)	101	47	68	69	69	68
6	Globin	46458	26	16	6(10)	144	107	119	84	90	100
7	Ferritin	47240	12	11	3(10)	259	125	137	77	91	100
8	SH3 domain	50044	14	7	2(10)	71	48	56	45	86	100
9	Lysozyme like	53955	9	8	10(51)	187	77	116	103	66	75
10	NAD(P) binding Rossmann fold	51735	49	6	3(44)	183	49	110	138	45	36

*Number(Number) => Number of structural entries (Total number of members in alignment.)
^Performance measured as (number of structurally equivalent residues reported by CUSP) *100/ (number of structurally equivalent residues reported by CE/CDD)

Table S4: Differences in number of protein structures [Helix, Strand and Coil: H,E,C] between longest and shortest members of length-rigid superfamilies

S.No	Code	Description	PDB_code	Number of sst			Hvar	Evar	Cvar
				H	E	C	(%)	(%)	(%)
1	47576	Calponin-homology domain,CH-domain	1aoa1	8	0	6	12.5	0	33.3
			1bkra	9	0	8			
2	48150	DNA-glycosylase	1mun	15	0	19	33.3	0	31.6
			1mpga	10	0	13			
3	48264	Cytochrome P450	1jpza	26	12	32	15.4	0	3.1
			1io7a	22	12	31			
4	48508	Nuclear receptor ligand-binding domain	2prga	14	3	16	7.1	33.3	6.2
			1qkma	13	2	15			
5	48576	Terpenoid synthases	1jfaa	23	0	22	30.4	0	9.1
			1di1a	16	0	20			
6	49373	Invasin/intimin cell-adhesion fragment	1cwva3	1	10	10	1	10	30
			1f00i2	0	11	13			
7	49562	C2 domain(Ca/lipid-bindingdomain,CaLB)	1k5wa	3	8	18	33.3	0	22.2
			1bdya	2	8	14			
8	49842	TNF-like	1jtzx	2	12	16	1	16.7	31.2
			1gr3a	0	10	11			
9	50182	Sm-like ribonucleoproteins	1d3bb	2	5	6	33.3	0	16.7
			1i8fa	3	5	5			
10	50405	ALDH-like	1dfca1	2	12	16	0	8.3	25
			1dfca4	2	11	12			
11	51206	cAMP-binding domain-like	1cx4a2	6	6	8	50	33.3	11
			1ft9a2	3	8	9			
12	53167	Purine and uridine phosphorylases	1b8oa	12	10	20	16.7	10	0
			1je0a	10	9	20			
13	53187	Zn-dependent exopeptidases	1lam 2	13	14	28	23.1	7.1	7.1
			1cg2a1	10	13	26			
14	53720	ALDH-like	1ez0a	24	18	36	8.3	11.1	2.8
			1k75a	22	16	37			
15	54117	Interleukin 8-like chemokines	1j9oa	2	3	7	0	0	14.3
			1qg7a	2	3	6			
16	54160	Chromo domain-like	1ap0	1	4	9	1	0	44.4
			1e0ba	2	4	5			
17	54334	Superantigen toxins, C-terminal domain	3seb 2	2	8	7	33.3	12.5	30
			3tss 2	3	7	10			
18	54495	UBC-like	2ucz	6	4	12	33.3	0	16.7
			1jatb	4	4	10			
19	54928	RNA-binding domain, RBD	1fj7a	2	4	8	0	0	12.5
			2msta	2	4	7			
20	55008	Metal-binding domain	1k0va	2	4	7	0	0	26.3
			1fe0a	2	4	4			
21	55979	DNA clamp	1dmla1	2	11	11	1	18.2	18.2
			1ge8a1	4	9	9			

**22**	**56281**	**Metallo-hydrolase/oxidoreductase**	**1smla**	**11**	**12**	**26**	**33.3**	**0**	**19.2**
			**2bc2a**	**6**	**12**	**21**			
**23**	**56327**	**Lactate&malate dehydrogenase,C-ter**	**7mdha2**	**8**	**8**	**14**	**12.5**	**12.5**	**21.4**
			**1hyha2**	**7**	**7**	**17**			
**24**	**56371**	**Ribosome inactivating proteins (RIP)**	**1dm0a**	**11**	**15**	**23**	**18.2**	**26.7**	**8.7**
			**1ce7a**	**9**	**11**	**21**			

Hvar, Evar, Cvar: Percentage variability in number of helices, strands and coils between the longest and shortest member of each superfamily

Table S5: Differences in number of protein structures [Helix,Strand and Coil: H,E,C] between longest and shortest members of length-deviant superfamilies

S.No	Code	Description	PDB_code	Number of sst			Hvar	Evar	Cvar
				H	E	C	(%)	(%)	(%)
1	46626	Cytochrome C	1iqca2	9	2	10	44.4	1	30
			1c75a-	5	0	7			
2	48179	6-phosphogluconate dehydrogenase C-terminal domain-like	1pgja1	15	2	16	66.7	1	81.2
			1dlja1	5	0	3			
3	49749	Viral proteins	1ruxa1	19	33	45	89.5	69.7	64.4
			1hx6a1	2	10	16			
4	51182	RmlC-like cupins	1pmi--	16	25	32	81.2	52	31.2
			1dgw-1	3	12	22			
5	53271	PRTase-like	1ecfa1	12	8	19	58.3	11.1	15.8
			1dkra2	5	9	16			
6	53067	Actin like ATPase domain	1bu6o1	13	15	21	53.8	46.7	28.6
			1j6za1	6	8	15			
7	53335	S-adenosyl-L-methionine-dependent methyltransferases	1f3la-	14	17	26	50	47.1	38.5
			1ej0a-	7	9	16			
8	53955	Lysozyme-like	1qusa-	15	7	18	60	42.9	38.9
			1iiza-	6	4	11			
9	56024	Phospholipase D/nuclease	1f0ia1	12	9	18	41.7	11.1	16.7
			1byra-	7	8	15			
10	49899	Concancavalin-A like lectins	1dypa	4	23	21	1	47.8	47.6
			1slta	0	12	11			
Hvar, Evar, Cvar: Percentage variability in number of helices, strands and coils between the longest and shortest member of each superfamily									
